# Capecitabine and mitomycin C as third-line therapy for patients with metastatic colorectal cancer resistant to fluorouracil and irinotecan

**DOI:** 10.1038/sj.bjc.6602733

**Published:** 2005-08-09

**Authors:** G Chong, J L B Dickson, D Cunningham, A R Norman, S Rao, M E Hill, T J Price, J Oates, N Tebbutt

**Affiliations:** 1 Department of Medicine, Royal Marsden Hospital, London and Surrey SM2 5PT, UK; 2 Department of Medical Oncology, The Queen Elizabeth and Lyell McEwin Hospitals, Woodville, Australia

**Keywords:** capecitabine, mitomycin C, colorectal cancer, chemotherapy resistance

## Abstract

Protracted venous infusion 5-fluorouracil (5FU) combined with mitomycin C (MMC) has demonstrated significant activity against metastatic colorectal cancer. Owing to potential synergy based upon upregulation of thymidine phosphorylase by MMC, the combination of capecitabine and MMC may improve outcomes in irinotecan-refractory disease. Eligible patients with progressive disease during or within 6 months of second-line chemotherapy were treated with capecitabine (1250 mg m^−2^ twice daily) days 1–14 every 3 weeks and MMC (7 mg m^−2^ IV bolus) once every 6 weeks. A total of 36 patients were recruited, with a median age of 64 years (range 40–77), and 23 patients (78%) were performance status 0–1. The objective response rate was 15.2%. In all, 48.5% of patients had stable disease. Median failure-free survival was 5.4 months (95% CI 4.6–6.2). Median overall survival was 9.3 months (95% CI: 6.9–11.7). Grade 3 toxicities were palmar-plantar erythema 16.7%, vomiting 8.3%, diarrhoea 2.8%, anaemia 8.3%, and neutropenia 2.8%. No patients developed haemolytic uraemic syndrome. Symptomatic improvement occurred for pain, bowel symptoms, and dyspnoea. Capecitabine in combination with MMC is an effective regimen for metastatic colorectal cancer resistant to 5FU and irinotecan with an acceptable toxicity profile and a convenient administration schedule.

Colorectal cancer (CRC) is one of the most common tumours worldwide, with approximately 1 million new cases diagnosed in 2000 accounting for over 500 000 deaths. At some stage during the course of the disease, 40–50% of patients develop metastatic disease. While effective agents are available for the first- and second-line treatment of metastatic CRC, there are relatively few published data on third-line therapies.

Capecitabine is a fluoropyrimidine prodrug that is metabolised to 5-fluorouracil (5FU) in a three-step process (Ishikawa *et al*, 1998). The last step requires thymidine phosphorylase, which is significantly more active in tumour compared to normal tissues. Hence, the conversion of capecitabine to active metabolite occurs preferentially at tumour sites. This may partially explain the differing toxicity profile compared to intravenous 5FU. Capecitabine monotherapy has been shown in randomised studies to be at least equivalent to bolus 5FU in terms of response rate, time to tumour progression, and overall survival (OS) for previously untreated metastatic CRC (Hoff *et al*, 2001; Van Cutsem *et al*, 2001). Owing to greater patient convenience, capecitabine has been substituted for short-duration bolus/infusion 5FU/leucovorin (LV5FU2) in combination schedules of oxaliplatin and irinotecan (Cassidy *et al*, 2004; Jordan *et al*, 2004).

Mitomycin C (MMC) is an antitumour antibiotic that has modest single-agent activity in patients with metastatic CRC (Hartmann *et al*, 1998; Anderson *et al*, 1999). A randomised study of 320 patients with untreated advanced CRC compared protracted venous infusion of 5FU (PVI 5FU) plus MMC and circadian-timed infusion of 5FU plus MMC (Price *et al*, 2004). This demonstrated a response rate of 38% and a median survival of 15.8 months in the PVI 5FU plus MMC arm. A previous study of 200 patients randomised to PVI 5FU plus MMC or PVI 5FU alone demonstrated an improved response rate in the combination arm (54 vs 38%, *P*=0.024) (Ross *et al*, 1997). However, there was no difference in OS. Another study of 24 patients with advanced CRC described a 12.5% response rate and median survival of 9.0 months using second-line PVI 5FU and MMC (Chester *et al*, 2000).

There is *in vivo* evidence of MMC-induced upregulation of intratumoral thymidine phosphorylase, which is the critical enzyme for the conversion of capecitabine to 5FU (Sawada *et al*, 1998). Hence, there is the possibility of clinically significant synergy between capecitabine and MMC. The capecitabine/MMC combination has been tested in patients with previously untreated metastatic CRC. A phase II study evaluated this combination in 92 first-line patients (Rao *et al*, 2004). Of 84 patients evaluable for response, the objective response rate was 38%, with a median OS of 14.3 months. The most frequent grade 3/4 toxicity was palmar-plantar erythema, which occurred in 19.7% of patients. There is limited data available on the capecitabine/MMC combination in patients with pretreated mestatatic CRC. A dose-escalation study enrolled 25 patients with pretreated advanced CRC (Hofheinz *et al*, 2004). Two patients (8%) achieved partial responses and the median progression-free survival was 2.0 months.

A significant proportion of patients with metastatic CRC are eligible for further chemotherapy following failure of two previous regimens. Given the encouraging efficacy and tolerability of capecitabine plus MMC in patients with treatment-naïve metastatic CRC, and the relative lack of data in pretreated patients, we conducted a phase II study in patients who had previously received two lines of chemotherapy for metastatic disease.

## PATIENTS AND METHODS

We conducted a phase II open-label study between July 2001 and November 2003 at two centres, one in the UK and one in Australia. The study was approved by both local medical ethics committees. Written, informed consent was obtained from each patient.

### Eligibility criteria

Inclusion criteria included histologically proven advanced or metastatic, inoperable adenocarcinoma of the colon or rectum, documented disease progression during or within 6 months following treatment with both 5FU and irinotecan, bidimensionally measurable disease as assessed by computed tomography (CT) scanning outside any previously irradiated area; ECOG performance status 0, 1, or 2; absence of any uncontrolled, concurrent medical conditions other than nonmelanotic skin cancer or carcinoma *in situ* of the uterine cervix, and life expectancy of greater than 3 months. Adequate baseline organ function was defined as follows: bone marrow (platelets >100 × 10^9^ l^−1^, WBC >3 × 10^9^ l^−1^, neutrophils >1.5 × 10^9^ l^−1^), renal (creatinine clearance >30 ml min^−1^), and hepatic (serum total bilirubin <1.5 × upper limit of normal range). Before entry into the study, all patients were required to have a CT scan of the chest, abdomen, and pelvis and carcinoembryonic antigen (CEA) measurement.

### Chemotherapy

The selected chemotherapy regimen was identical to that used in a previous study which demonstrated safety and efficacy in previously untreated patients with advanced CRC (Rao *et al*, 2004).

Mitomycin C was delivered as an intravenous bolus at a dose of 7 mg m^−2^ every 6 weeks. Capecitabine (2500 mg m^−2^ day^−1^) was administered orally in two divided doses for 14 days followed by a 7-day treatment-free interval. Each capecitabine cycle was repeated every 21 days. Patients continued therapy for 12 weeks and were then reassessed. If there was no evidence of disease progression, treatment was then continued for a further 12 weeks. The maximum duration of therapy was 24 weeks.

### Toxicity evaluation and dose modification

Toxicity was evaluated and graded according to the National Cancer Institute Common Toxicity Criteria (Version 2.0). For grade 3 nonhaematological toxicity, capecitabine therapy was suspended until resolution and reinitiated with a 25% dose reduction for the first occurrence and 50% for the second. For grade 4 nonhaematological toxicity, capecitabine therapy was either terminated or suspended until resolution, with a 50% dose reduction upon reinitiation at the treating physician's discretion. For haematological toxicity, if the absolute neutrophil count was less than 1.0 × 10^9^ l^−1^ or the platelet count was less than 100 × 10^9^ l^−1^, capecitabine and MMC were delayed until resolution and reinitiated at full dose for a 1-week delay or with a 25% dose reduction for a 2-week delay.

### Efficacy evaluation

Tumour response was investigator-assessed by CT scan according to RECIST criteria at 12 and 24 weeks. Eastern Co-operative Oncology Group performance status (PS) was assessed at baseline, 12 weeks, and 24 weeks after commencement of chemotherapy and every 3 months thereafter until death or disease progression. Failure-free survival (FFS) and OS were calculated for all patients from the date of treatment commencement. Failure-free survival was defined as time to tumour progression or death from any cause. Overall survival was defined as time to death from any cause. The method of Kaplan and Meier was used. Symptoms of dysphagia, reflux, pain, anorexia, weight loss, nausea, vomiting, altered bowel habit, lethargy, and dyspnoea were prospectively documented as present or absent at baseline and throughout the treatment period. Symptoms that resolved from present to absent during the course of therapy were coded as symptomatic response for that parameter.

### Statistical methods

This phase II study was constructed using the Simon optimal two-stage design. The sample size was calculated with 90% power to detect an objective response rate of 20% and to rule out a response rate of 5% using a one-sided test. The first stage was determined to be 21 patients, after which an interim analysis was performed. The criterion for continued accrual was the observation of at least one tumour response. The second stage was planned to accrue a further 20 patients provided this condition was met. The study was terminated after enrolment of 36 patients due to slowing accrual.

## RESULTS

A total of 36 patients were entered into the study between July 2001 and November 2003. Baseline patient characteristics are presented in Table 1. The median age was 64 (range 40–77) years. In all, 78% of patients had a PS of 0 or 1. All patients had received irinotecan as either first- or second-line therapy. Only two patients had received prior oxaliplatin. No patient had received prior capecitabine.

### Chemotherapy delivery

The median treatment duration was 18.5 weeks (range 1–28). The median dose intensity for capecitabine was 81.5% of the starting dose.

### Tumour and symptomatic response

A total of 33 patients were evaluable for response. Three patients who withdrew from the study for logistical reasons prior to response assessment were not evaluable for response. The overall response rate was 15.2%. In all, 48.5% had stable disease. Improvement in tumour-related symptoms was observed in a substantial proportion of patients who had baseline pain, lethargy, dyspnoea, altered bowel habit, anorexia, or weight loss (Tables 2 and 3).

### Toxicity

Capecitabine/MMC was a well-tolerated regimen with no grade 4 toxicity experienced by any patient. All grade 3 toxicities are presented in Table 4. Grade 3 haematological toxicities were anaemia (8.3%), neutropenia (2.8%), and thrombocytopenia (2.8%). The most frequent grade 3 nonhaematological toxicities were palmar-plantar erythema (16.7%), nausea/vomiting (8.3%), and lethargy (5.6%). Two patients (5.6%) developed grade 2 chest pain thought to be related to capecitabine and were therefore taken off study. One patient developed tumour-related hydronephrosis; therefore MMC was ceased. There were no cases of red cell fragmentation requiring cessation of MMC, and no cases of haemolytic uraemic syndrome were observed.

### Survival

**Figure 1:**
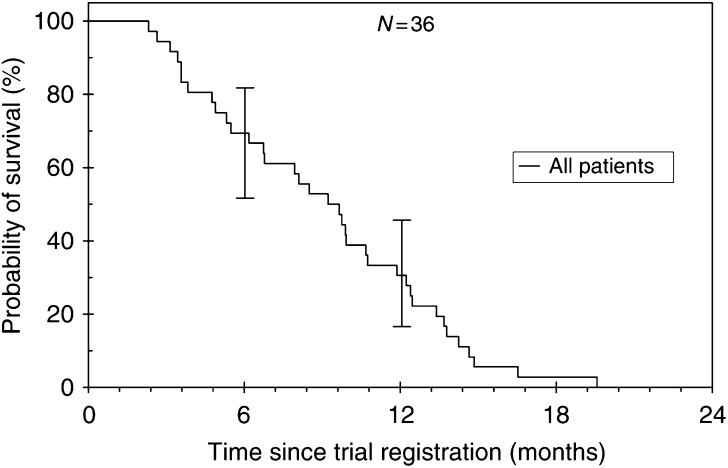
Survival.

**Figure 2:**
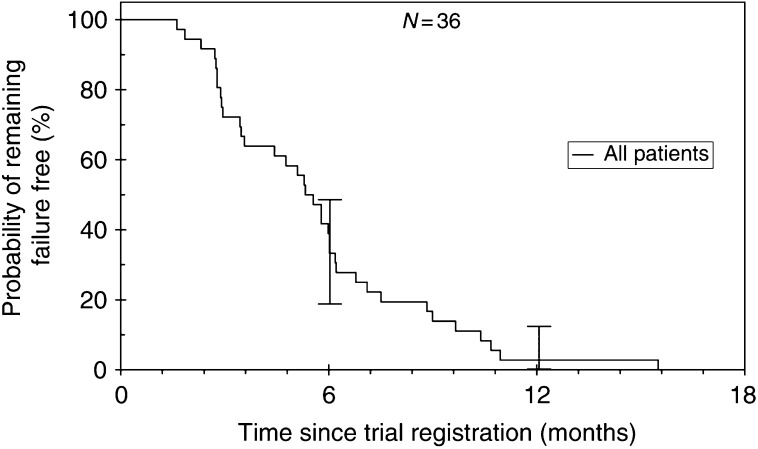
Failure-free survival.

At the time of analysis, survival data were complete, with no surviving patients. The median overall survival was 9.3 months (95% CI: 6.9–11.7) with a 1-year survival of 30.6% (Figure 1). The median FFS was 5.4 months (95% CI: 4.6–6.2) (Figure 2). A total of 11 patients went on to receive fourth-line treatment following completion of this study; eight received oxaliplatin.

## DISCUSSION

The combination of capecitabine and MMC is efficacious, results in symptom relief and is well tolerated as third-line treatment of metastatic CRC. This is consistent with the observed efficacy of the same regimen when administered to patients with untreated advanced CRC (Rao *et al*, 2004). The demonstrated activity of capecitabine/MMC in this study contrasts with the lack of response to capecitabine monotherapy seen in a phase II study of 5FU-refractory advanced colorectal cancer (Hoff *et al*, 2004). Capecitabine/MMC therefore represents a plausible regimen for those patients who are eligible for treatment after failure of two previous regimens. In the current era of targeted therapies, capecitabine/MMC may be a reasonable alternative if targeted therapies such as cetuximab are unavailable or contraindicated.

The observed objective response rate of 15.2% and median overall survival of 9.3 months in this study compares favourably with other trials of patients with advanced CRC previously treated with 5FU and irinotecan. Kemeny *et al* performed a randomised study in which 214 patients who had previously received sequential 5FU and irinotecan received either bolus/infused 5FU/LV (LV5FU2) or oxaliplatin/LV5FU2 (FOLFOX4) as third-line treatment for advanced CRC. An objective response rate of 13% and a median survival of 9.9 months were found in patients receiving FOLFOX4 (Kemeny *et al*, 2004). The response rate in patients receiving LV5FU2 was only 2%. However, the median survival (11.4 months, *P*=0.2) was similar for patients randomised to receive LV5FU2, which could have been due to protocol-permitted crossover to FOLFOX4 in patients with progressive disease on LV5FU2. In fact, 69% of patients in the LV5FU2 arm subsequently received oxaliplatin upon tumour progression.

These data are important for the interpretation of our capecitabine/MMC median survival, as they imply that whether oxaliplatin was given third- or fourth-line, the OS was similar. Therefore, although only 22% of our patients went on to subsequent fourth-line oxaliplatin, the median survival of patients receiving capecitabine/MMC third-line was consistent with that observed for oxaliplatin or LV5FU2 given third-line in the Kemeny study. Tumour response rates were also similar for capecitabine/MMC and FOLFOX4 across the two studies. One potential advantage, however, of delaying oxaliplatin use may be to reduce the impact of oxaliplatin-induced sensory neuropathy.

Of note, the capecitabine/MMC regimen used in our cohort appears to have similar or greater efficacy than the combination of raltitrexed and MMC given as third-line treatment for metastatic CRC. A study of 21 patients who had all previously received 5FU, irinotecan, and oxaliplatin were treated with raltitrexed plus MMC on a 4-week schedule (Rosati *et al*, 2003). Of 16 patients evaluable for response, there were no objective tumour responses, although 33.6% had stable disease. The median overall survival was 5 months. While the patients in our study had generally not received oxaliplatin as first- or second-line treatment, as discussed above, the order in which a patient receives cytotoxic agents may not impact on OS times.

Another study of capecitabine/MMC in patients with oxaliplatin and irinotecan-refractory advanced colorectal cancer supports the activity of this combination as third-line therapy. In total, 20 response-evaluable patients were treated with capecitabine 2000 mg m^−2^ days 1–14 every 3 weeks and MMC 7 mg m^−2^ every 6 weeks (Harba *et al*, 2003). A total of 10% achieved a partial response and the median OS was 7.75 months. Of note, the capectabine dose was 20% lower than in our study. Another study using the same schedule as Harba *et al* also demonstrated a 4.8% response rate and median survival of 6.8 months in the third-line setting (Lim do *et al*, 2005).

Therapies targeting signalling transduction pathways such as the epidermal growth factor receptor (EGFR) pathway are currently being studied in advanced CRC. The BOND study randomised 329 EGFR-expressing irinotecan-refractory patients to irinotecan and cetuximab or cetuximab alone (Cunningham *et al*, 2004). The majority of patients had received two or more previous lines of chemotherapy, although 20.7% had only received one previous chemotherapy regimen. While the response rate in patients receiving combination therapy was 22.9%, that for the cetuximab monotherapy arm was 10.8%. Overall survival was 8.6 months in the combination arm and 6.9 months in the monotherapy arm. It is interesting to note that in our study, capecitabine/MMC appeared to have comparable response rate to cetuximab monotherapy in patients pretreated with 5FU followed by irinotecan.

The relatively low toxicity experienced by patients receiving capecitabine and MMC is attractive for patients eligible for third-line therapy for metastatic CRC. Patients often have substantial cumulative toxicities from previous chemotherapy, and may be of poorer performance status than those eligible for first-line therapy. Additional toxicity from MMC was essentially limited to greater myelosuppression than would be expected for capecitabine monotherapy. However, no episodes of grade 3 infection were observed. There were no cases of haemolytic uraemic syndrome observed in our cohort all of whom had normal baseline renal function. The acceptable toxicity profile is reinforced by the observation that 11 patients were fit enough subsequently to go onto fourth-line therapy.

In conclusion, the combination of capecitabine and MMC is active and well tolerated as third-line therapy in patients who are refractory to irinotecan. Our study has demonstrated tumour response rate and survival data that are comparable to other third-line therapeutic options, such as oxaliplatin or cetuximab-based regimens. Capecitabine and MMC could therefore be considered as a third-line treatment option if patients are not eligible for treatment with these regimens.

## Figures and Tables

**Table 1:** Patient characteristics

**Patient characteristics**	**Number (%)**
Total enrolled	36
Median age (years) (range)	64 (40–77)
Male : female	21 : 15
	
*ECOG performance status*	
0	10 (27.8)
1	18 (50.0)
2	8 (22.2)
	
*Primary site*	
Colon	27 (75)
Rectum	9 (25)
	
*Sites of metastases*	
Liver	31 (86.1)
Lung	14 (38.9)
Nodal	6 (16.7)
Peritoneum	6 (16.7)
Locoregional	5 (13.9)
	
*Histological differentiation*	
Moderate	29 (80.6)
Poor	4 (11.1)
Unknown	3 (8.3)
	
*Previous first-line therapies*	
5-Fluorouracil	35
UFT	1
Oxaliplatin	2
Irinotecan	1
	
*Previous second-line therapies*	
Irinotecan	35
Cetuximab	1

**Table 2:** Tumour response to capecitabine/MMC

**Best response**	**Number of patients**	**%**
CR	0	0
PR	5	15.2
SD	16	48.5
PD	12	36.4
NE	3	

CR=complete response; PR=partial response; SD=stable disease; PD=progressive disease; NE=not evaluable.

**Table 3:** Symptom response to capecitabine/MMC

**Symptom**	**Proportion of patients with baseline symptoms resolving on therapy**	**%**
Pain	14/18	77.8
Lethargy	9/22	40.9
Altered bowel habit	7/10	70
Dyspnoea	5/6	83.3
Anorexia	3/6	50
Weight loss	2/4	50

**Table 4:** Toxicity

**Toxicity**	**All grades (*n*=36) (%)**	**Grade 3 (%)**
Palmar-plantar erythema	50	16.7
Nausea/vomiting	44.5	8.3
Lethargy	94.5	5.6
Diarrhoea	52.8	2.8
Peripheral neuropathy	8.3	2.8
Fever	8.6	2.8
Stomatitis	33.3	0
Infection	11.1	0
		
Anaemia	61.1	8.3
Neutropenia	11.1	2.8
Thrombocytopenia	16.7	2.8

## References

[bib1] Anderson N, Lokich J, Moore C, Bern M, Coco F (1999) A dose-escalation phase II clinical trial of infusional mitomycin C for 7 days in patients with advanced measurable colorectal cancer refractory or resistant to 5-fluorouracil. Cancer Invest 17: 586–593 10592766 10.3109/07357909909032844

[bib2] Cassidy J, Tabernero J, Twelves C, Brunet R, Butts C, Conroy T, Debraud F, Figer A, Grossmann J, Sawada N, Schoffski P, Sobrero A, Van Cutsem E, Diaz-Rubio E (2004) XELOX (capecitabine plus oxaliplatin): active first-line therapy for patients with metastatic colorectal cancer. J Clin Oncol 22: 2084–2091 15169795 10.1200/JCO.2004.11.069

[bib3] Chester JD, Dent JT, Wilson G, Ride E, Seymour MT (2000) Protracted infusional 5-fluorouracil (5-FU) with bolus mitomycin in 5-FU-resistant colorectal cancer. Ann Oncol 11: 235–237 10.1023/a:1008356017611 10761764

[bib4] Cunningham D, Humblet Y, Siena S, Khayat D, Bleiberg H, Santoro A, Bets D, Mueser M, Harstrick A, Verslype C, Chau I, Van Cutsem E (2004) Cetuximab monotherapy and cetuximab plus irinotecan in irinotecan-refractory metastatic colorectal cancer. N Engl J Med 351: 337–345 15269313 10.1056/NEJMoa033025

[bib5] Harba A, Jordan K, Kegel T, Behrens R, Grothey A, Schmoll HJ (2003) Capecitabine/mitomycin C as salvage therapy in oxaliplatin and CPT11 refractory advanced colorectal carcinoma. Proc Am Soc Clin Oncol 22: (abstr. 1335) p332

[bib6] Hartmann JT, Harstrick A, Daikeler T, Kollmannsberger C, Muller C, Seeber S, Kanz L, Bokemeyer C (1998) Phase II study of continuous 120 h infusion of mitomycin C as salvage chemotherapy in patients with progressive or rapidly recurrent colorectal cancer. Anticancer Drugs 9: 427–431 9660540 10.1097/00001813-199806000-00009

[bib7] Hoff PM, Ansari R, Batist G, Cox J, Kocha W, Kuperminc M, Maroun J, Walde D, Weaver C, Harrison E, Burger HU, Osterwalder B, Wong AO, Wong R (2001) Comparison of oral capecitabine *vs* intravenous fluorouracil plus leucovorin as first-line treatment in 605 patients with metastatic colorectal cancer: results of a randomized phase III study. J Clin Oncol 19: 2282–2292 11304782 10.1200/JCO.2001.19.8.2282

[bib8] Hoff PM, Pazdur R, Lassere Y, Carter S, Samid D, Polito D, Abbruzzese JL (2004) Phase II study of capecitabine in patients with fluorouracil-resistant metastatic colorectal carcinoma. J Clin Oncol 22: 2078–2083 15169794 10.1200/JCO.2004.05.072

[bib9] Hofheinz RD, Hartmann JT, Willer A, Oechsle K, Hartung G, Gnad U, Saussele S, Kreil S, Bokemeyer C, Hehlmann R, Hochhaus A (2004) Capecitabine in combination with mitomycin C in patients with gastrointestinal cancer: results of an extended multicentre phase-I trial. Br J Cancer 91: 834–838 15238990 10.1038/sj.bjc.6602025 PMC2409860

[bib10] Ishikawa T, Utoh M, Sawada N, Nishida M, Fukase Y, Sekiguchi F, Ishitsuka H (1998) Tumor selective delivery of 5-fluorouracil by capecitabine, a new oral fluoropyrimidine carbamate, in human cancer xenografts. Biochem Pharmacol 55: 1091–1097 9605432 10.1016/s0006-2952(97)00682-5

[bib11] Jordan K, Kellner O, Kegel T, Schmoll HJ, Grothey A (2004) Phase II trial of capecitabine/irinotecan and capecitabine/oxaliplatin in advanced gastrointestinal cancers. Clin Colorectal Cancer 4: 46–50 15207020 10.3816/ccc.2004.n.009

[bib12] Kemeny N, Garay CA, Gurtler J, Hochster H, Kennedy P, Benson A, Schwab BD, Polikoff J, Wertheim M, Shumaker G, Hallman D, Burger B, Gupta S (2004) Randomized multicenter phase II trial of bolus plus infusional fluorouracil/leucovorin compared with fluorouracil/leucovorin plus oxaliplatin as third-line treatment of patients with advanced colorectal cancer. J Clin Oncol 22: 4701–4709 10.1200/JCO.2004.03.119 15570076

[bib13] Lim do H, Park YS, Park BB, Ji SH, Lee J, Park KW, Kang JH, Lee SH, Park JO, Kim K, Kim WS, Jung CW, Im YH, Kang WK, Park K (2005) Mitomycin-C and capecitabine as third-line chemotherapy in patients with advanced colorectal cancer: a phase II study. Cancer Chemother Pharmacol 56: 10–14 10.1007/s00280-004-0963-2 15782313

[bib14] Price TJ, Ross PJ, Hickish T, Tait D, Norman AR, Ford HE, Middleton G, Sumpter K, Hill M, Oates J, Cunningham D (2004) Phase III study of mitomycin-C with protracted venous infusion or circadian-timed infusion of 5-fluorouracil in advanced colorectal carcinoma. Clin Colorectal Cancer 3: 235–242 15025796 10.3816/CCC.2004.n.004

[bib15] Rao S, Cunningham D, Price T, Hill ME, Ross PJ, Tebbutt N, Norman AR, Oates J, Shellito P (2004) Phase II study of capecitabine and mitomycin C as first-line treatment in patients with advanced colorectal cancer. Br J Cancer 91: 839–843 15266319 10.1038/sj.bjc.6602039 PMC2409883

[bib16] Rosati G, Rossi A, Germano D, Reggiardo G, Manzione L (2003) Raltitrexed and mitomycin-C as third-line chemotherapy for colorectal cancer after combination regimens including 5-fluorouracil, irinotecan and oxaliplatin: a phase II study. Anticancer Res 23: 2981–2985 12926149

[bib17] Ross P, Norman A, Cunningham D, Webb A, Iveson T, Padhani A, Prendiville J, Watson M, Massey A, Popescu R, Oates J (1997) A prospective randomised trial of protracted venous infusion 5-fluorouracil with or without mitomycin C in advanced colorectal cancer. Ann Oncol 8: 995–1001 9402173 10.1023/a:1008263516099

[bib18] Sawada N, Ishikawa T, Fukase Y, Nishida M, Yoshikubo T, Ishitsuka H (1998) Induction of thymidine phosphorylase activity and enhancement of capecitabine efficacy by taxol/taxotere in human cancer xenografts. Clin Cancer Res 4: 1013–1019 9563897

[bib19] Van Cutsem E, Twelves C, Cassidy J, Allman D, Bajetta E, Boyer M, Bugat R, Findlay M, Frings S, Jahn M, McKendrick J, Osterwalder B, Perez-Manga G, Rosso R, Rougier P, Schmiegel WH, Seitz JF, Thompson P, Vieitez JM, Weitzel C, Harper P (2001) Oral capecitabine compared with intravenous fluorouracil plus leucovorin in patients with metastatic colorectal cancer: results of a large phase III study. J Clin Oncol 19: 4097–4106 11689577 10.1200/JCO.2001.19.21.4097

